# LincRNA-p21 acts as a mediator of ING1b-induced apoptosis

**DOI:** 10.1038/cddis.2015.15

**Published:** 2015-03-05

**Authors:** U M Tran, U Rajarajacholan, J Soh, T-s Kim, S Thalappilly, C W Sensen, K Riabowol

**Affiliations:** 1 Department of Biochemistry and Molecular Biology, and Oncology, University of Calgary, 3330 Hospital Drive NW, Calgary, T2N 4N1 Alberta, Canada; 2 Department of Biochemistry and Molecular Biology, Visual Genomics Centre, University of Calgary, 3330 Hospital Drive NW, Calgary, T2N 4N1 Alberta, Canada

## Abstract

ING1b is a tumor suppressor that affects transcription, cell cycle control and apoptosis. ING1b is deregulated in disease, and its activity is closely linked to that of p53. In addition to regulating protein-coding genes, we found that ING1b also influences the expression of large intergenic non-coding RNAs (lincRNAs). In particular, lincRNA-p21 was significantly induced after DNA-damage stress or by ING1b overexpression. Furthermore, lincRNA-p21 expression in response to DNA damage was significantly attenuated in cells lacking ING1b. LincRNA-p21 is also a target of p53 and can trigger apoptosis in mouse cell models. We found that this function of lincRNA-p21 is conserved in human cell models. Moreover, ING1b and p53 could function independently to influence lincRNA-p21 expression. However, their effects become more additive under conditions of stress. In particular, ING1b regulates lincRNA-p21 levels by binding to its promoter and is required for induction of lincRNA-p21 by p53. The ability of ING1b to cause apoptosis is also impaired in the absence of lincRNA-p21. Surprisingly, deletion of the ING1b plant homeodomain, which allows it to bind histones and regulate chromatin structure, did not alter regulation of lincRNA-p21. Our findings suggest that ING1b induces lincRNA-p21 expression independently of histone 3 lysine 4 trimethylation mark recognition and that lincRNA-p21 functions downstream of ING1b. Thus, regulation at the level of lincRNA-p21 may represent the point at which ING1b and p53 pathways converge to induce apoptosis under specific stress conditions.

ING1b is a type II tumor suppressor that is downregulated in many cancers.^1, 2^ As it impacts the expression of genes that affect apoptosis, senescence and cell cycle regulation, reduction in ING1b could influence cell viability and disease progression by altering transcription.^1^ ING1b contains several conserved domains that act as binding regions for chromatin marks such as trimethylated histone H3 lysine 4 (H3K4me3), and chromatin-modifying complexes.^3, 4^ The plant homeodomain (PHD), in particular, binds H3K4me3 in a methylation-sensitive manner, allowing ING1b to act as a histone code ‘reader'.^5, 6, 7, 8^ Hence, ING1b can directly bind to promoters of certain genes and control their expression by altering surrounding chromatin structure.^9, 10^ However, the role of ING1b in other epigenetic mechanisms, especially through long non-coding RNAs is still not well understood.

The involvement of ING1b in apoptosis has been reported to be both dependent and independent of p53.^11, 12^ On one hand, without p53, ING1b could still induce expression of p21 and bax, which are proteins having significant effects on regulation of apoptosis and promotion of mitochondria-associated cell death, respectively.^13^ On the other hand, knocking out ING1b in mouse fibroblasts increased cell proliferation in both p53-positive and p53-null environments.^13^ DNA-damage-induced expression of another apoptotic protein, puma, by p53 was also unaffected by the absence of ING1b in mouse thymocytes.^14^ However, when both ING1b and p53 proteins are present, apoptosis following DNA damage can be significantly enhanced, suggesting that they are essential for an appropriate cell stress response.^13^ Although it is now clear that ING1b can stabilize p53 through several pathways, including prevention of proteasomal degradation,^15^ the functional relationship between ING1b and p53, especially at the gene regulation level, is still largely undefined.

In recent studies, large intergenic long non-coding RNA p21 (lincRNA-p21) has been shown to regulate apoptosis in response to p53 signaling and to facilitate suppression of target gene translation.^16, 17^ In addition, lincRNA-p21 stability can be influenced by RNA-binding protein HuR.^17^ In this study, we find evidence to support the idea that ING1b also transcriptionally regulates lincRNA-p21. This effect requires domains in the amino terminus of ING1b but appears to be independent of the ING1b PHD or the presence of HuR. Moreover, lincRNA-p21 expression is crucial for ING1b-induced apoptosis in response to DNA-damage stress. This effect is significantly enhanced by p53. Thus, lincRNA-p21 may represent the point at which ING1b and p53 pathways converge to induce apoptosis in cells.

## Results

### LincRNA-p21 levels increase in response to ING1b overexpression

**Figure 1:**
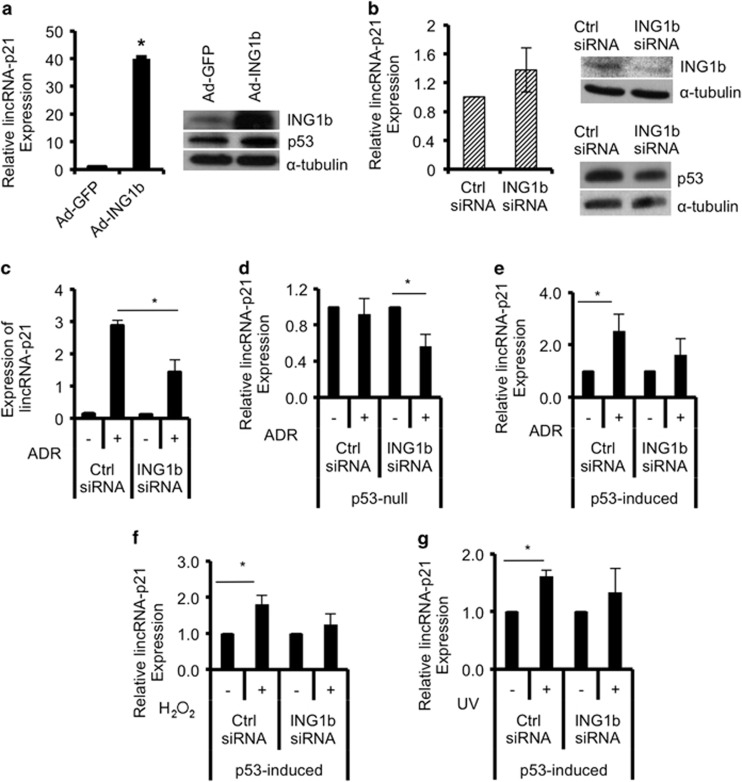
ING1b regulates lincRNA-p21 expression, and both ING1b and p53 are required for lincRNA-p21 induction after DNA damage. (**a**) Following infection of normal human diploid fibroblasts (Hs68) with adenoviral constructs containing GFP (Ad-GFP) or GFP-ING1b (Ad-ING1b), expression of lincRNA-p21 was measured using qRT-PCR, normalized to *β*-actin and graphed relative to Ad-GFP controls. ING1b, p53 and *α*-tubulin protein levels were examined using western blot analysis. (**b** and **c**) Hs68 cells were transfected with control or ING1b siRNAs for 32 h, and a subset was treated with Adriamycin (ADR) for another 16 h. RNA levels of lincRNA-p21 were measured using qRT-PCR, normalized to GAPDH and graphed relative to controls. ING1b and p53 protein levels were determined using western blot and normalized to *α*-tubulin protein expression. P53-null and p53-induced H1299 cells were transfected with ING1b or control siRNAs for 24 h before treatment with 500 nM ADR for 16 h (**d** and **e**), 100 *μ*M hydrogen peroxide (H_2_O_2_) for 24 h (**f**) and 85 J/m^2^ of UV (24 h) (**g**). RNA was extracted and lincRNA-p21 was quantified using qRT-PCR. All values in this figure represent an average of three independent experiments (asterisks indicate *P*<0.05)

Previous studies have reported that ING1b-induced apoptosis can be either p53-dependent or p53-independent, with variations being contingent on cell type.^15^ Moreover, p53 was shown to require ING1 for induction of programmed cell death,^11, 13, 14^ suggesting the potential for a functional interdependency between ING1 and p53. Recently, lincRNA-p21 was shown to be a transcriptional target of p53 that mediates gene repression to promote apoptosis.^16^ Given this relationship between ING1 and p53, we asked if ING1b could influence lincRNA-p21 expression. ING1b was overexpressed using an adenoviral construct encoding GFP and ING1b expressed from different promoters, or knocked down with siRNAs in Hs68 human diploid fibroblasts. Levels of lincRNA-p21 was subsequently examined using quantitative real-time polymerase chain reactions (qRT-PCR). In this human diploid fibroblast strain, ING1b increased lincRNA-p21 levels by ~40-fold (Figure 1a). In contrast, lincRNA-p21 did not decrease in response to ING1b knockdown (Figure 1b), suggesting that ING1b influences lincRNA-p21 expression only at levels that induce cell stress, similar to those during apoptosis.^18, 19^ As lincRNA-p21 was also reported to respond to p53 downstream of DNA-damage signals,^16^ we next tested the effects of ING1b on lincRNA-p21 in the presence of damage. Hs68s treated with control oligo or with siING1b were exposed to adriamycin (ADR) to induce DNA damage. Under control conditions, lincRNA-p21 expression increased significantly after DNA damage (Figure 1c, Supplementary Figure S1A), but induction was only 50% as efficient when ING1b was knocked down (Figure 1c, *P*<0.05), suggesting ING1b was transducing a major part of the ADR stress signal. This was not due to the effects of ING1b on p53 transcription (Supplementary Figure S1B). Instead, we found that this decrease in efficiency but not absolute suppression of lincRNA-p21 expression may be due to an increase in the half-life of lincRNA-p21 after ING1b knockdown. Another factor that might affect the efficiency of knockdown might be differential stabilization of ING1 protein following DNA damage as reported previously for conditions of oxidative stress.^20, 21^ Compared with control, cells treated with ING1b shRNA exhibited increased lincRNA-p21 stability after DNA damage (Supplementary Figure S1C). In this experiment actinomycin D (ActD) was used to block ongoing transcription to estimate the *t*_1/2_ of lincRNA-p21. As ActD functions by binding gene promoters to block transcription and ING1b also binds the promoter region of lincRNA-p21 (see below), knockdown of ING1b might also interfere with the ability of ActD to block transcription, leading to apparent increased lincRNA-p21 stability.

### ING1b and p53 are required for lincRNA-p21 induction after DNA damage

In human non-small cell lung carcinoma cells that are p53-null, ADR failed to induce expression of lincRNA-p21. In these cells, ING1b knockdown by ~80% (Supplementary Figure S1D) also drove lincRNA-p21 expression downward (Figure 1d). In cells inducibly expressing p53, it was apparent that DNA damage could cause lincRNA-p21 induction. However, the effect was again diminished when ING1b was knocked down (Figure 1e, Supplementary Figure S1D), indicating that both ING1b and p53 are required for maximal response to DNA damage through lincRNA-p21, especially in lung carcinoma. Of note, p53 expression increased when ADR was present in both experiments (Supplementary Figure S1D panel 3), yet lincRNA-p21 could still be significantly affected by ING1b knockdown, further indicating that ING1 has a role in lincRNA-p21 regulation.

**Figure 2:**
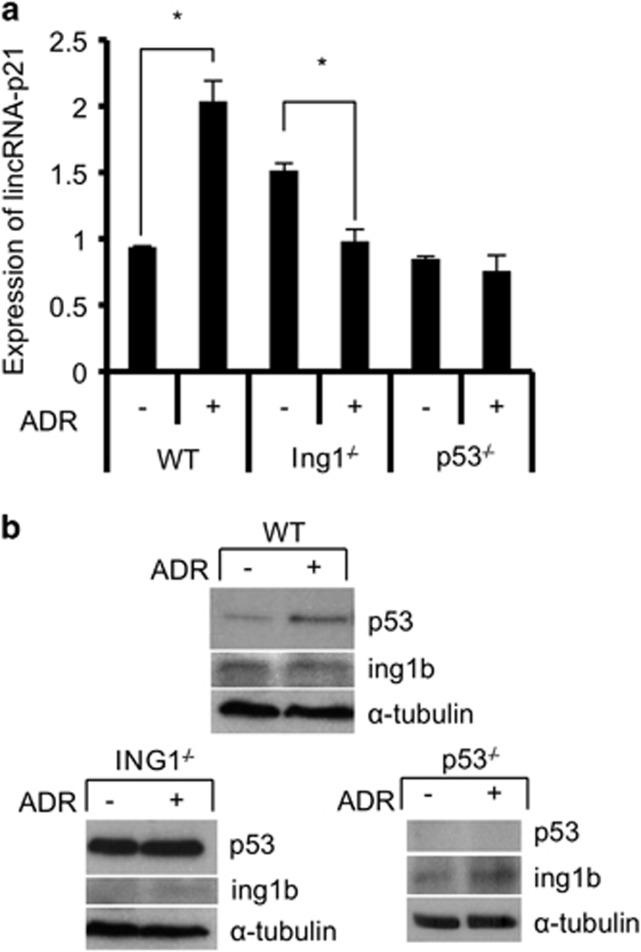
ING1b and p53 are also required for lincRNA-p21 induction in the mouse fibroblast cell model. (**a**) Wild-type, ING1^−/−^ and p53^−/−^ mouse embryonic fibroblasts (MEF) were either exposed to 500 nM ADR for 16 h (+) or left untreated (−). RNA level of lincRNA-p21 was measured and normalized to histone H3 levels. Treated samples were subsequently compared with untreated controls. (**b**) P53, ING1b and *α*-tubulin protein levels were measured using western blot analysis. All values in this figure represent an average of three independent experiments (asterisks indicate that *P*<0.05)

To determine whether other types of DNA damage had similar effects, human lung cancer cells were treated with either hydrogen peroxide (H_2_O_2_) or UV and tested for lincRNA-p21 expression. Results indicated that like ADR, H_2_O_2_ and UV increased lincRNA-p21 levels but knocking down ING1b had a less significant effect on lincRNA-p21 expression (Figures 1f and 1g). The impact of ING1b on lincRNA-p21 in response to H_2_O_2_ and UV also appeared to be unaffected by changing levels of p53 (Supplementary Figures S1E and F). The above data are consistent with ING1b and p53 having a major effect on lincRNA-p21 regulation, although the magnitude of influence varies with the pathways activated by different forms of stress. As knockdown cannot eliminate ING1b or p53, we treated wild-type, ING1 knockout (ING1^−/−^) or p53 knockout (p53^−/−^) mouse embryo fibroblasts (MEFs) with ADR and compared lincRNA-p21 expression in different treatment groups. As shown in Figure 2, lincRNA-p21 levels increased significantly after treatment of wild-type cells. However, this was not seen after DNA damage in ING1 or p53 knockout MEFs (Figure 2). At the protein level, p53 was stabilized by ADR (Figure 2) and compared with wild-type MEFs, p53 was significantly higher in ING1 knockout MEFs, which may contribute to the baseline increase in lincRNA-p21 in untreated knockout cells. In addition, bax, a pro-apoptotic gene known to be a target of p53^22^ and ING1b,^13^ also had a similar pattern of expression as lincRNA-p21 (Supplementary Figure S2A), suggesting that they may be regulated by similar pathways. These experiments suggest that when expression of either p53 or ING1 is compromised, the effect of ADR on lincRNA-p21 expression is blunted.

### ING1b and p53 independently affect lincRNA-p21 induction in response to stress

**Figure 3:**
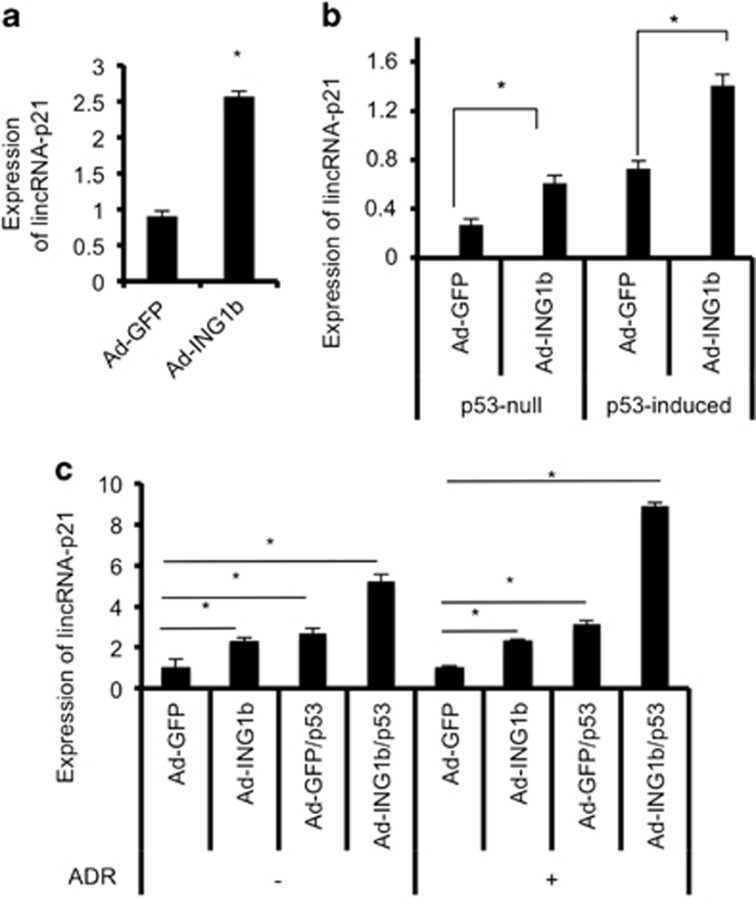
ING1b and p53 induce lincRNA-p21 expression in an additive manner. (**a**) Human liver cancer cells (HEP3B, p53-null) were infected with either GFP (Ad-GFP) or ING1b (Ad-ING1b) viruses for 24 h. The RNA levels of lincRNA-p21 were determined using qRT-PCR and normalized to GAPDH. (**b** and **c**) Human non-small cell lung cancer carcinoma cells (H1299, p53-null) were infected with either GFP or ING1b viruses for 6 h. Subsequently, 100 ng/ml of doxycycline (DOX) was added to the media to induce the expression of p53. At 14 h, one group was treated with 500 nM of ADR. The cells were then left in an incubator for an additional 16 h before sample collection. RNA expression of lincRNA-p21 was measured using qRT-PCR and normalized to GAPDH levels. ADR-negative or ADR-positive treatment groups were compared with their respective Ad-GFP sample. All experimental values were derived from three replicates (asterisks indicate that *P*<0.05)

ING1b overexpression stabilized and increased p53 activity (Figure 1a)^15, 23^ and lincRNA-p21 promoter contains p53-binding motifs.^16^ Thus, we asked whether the increase in lincRNA-p21 after ING1b overexpression was an indirect consequence of p53 stabilization by ING1b. To this end, we measured lincRNA-p21 levels after ING1b overexpression in conditions with or without p53 to determine whether ING1b could upregulate lincRNA-p21 independently of p53. In two different p53-null cell lines, we found that lincRNA-p21 increased after infection of cells with GFP-ING1b adenoviruses (Figures 3a and b,Supplementary Figure S2B). However, levels of lincRNA-p21 were approximately twice as high when both ING1b and p53 were present (Figure 3b). Of note, as opposed to results in human cells, p53 appears to be necessary for significant ING1b-induced expression of lincRNA-p21 in p53^−/−^ MEF cells (Supplementary Figure S2C). In cells not treated with ADR, overexpression of ING1b and p53 together drove lincRNA-p21 expression up approximately twofold greater than when either protein was overexpressed alone (Figure 3c), and this effect was enhanced in the presence of ADR. Thus, the above experiments indicate that especially in human cells, ING1b can act independently of p53 to increase lincRNA-p21, but when both proteins are present under stress, their effects become additive.

### ING1b regulates lincRNA-p21 levels independently of HuR

**Figure 4:**
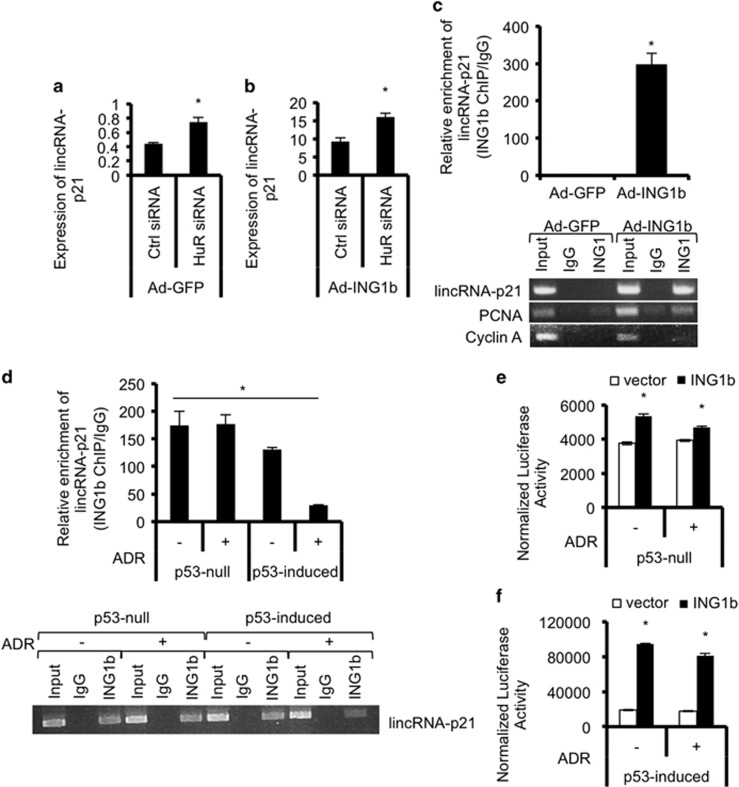
ING1b directly binds to the lincRNA-p21 promoter and regulates its expression at the transcriptional level. (**a** and **b**) The effects of ING1b on lincRNA-p21 expression are independent of HuR, which regulates lincRNA-p21 stability. lincRNA-p21 expression was measured using qRT-PCR after Hs68 cells were transfected with control or HuR siRNAs for 8 h and treated with either Ad-GFP or Ad-ING1b for an additional 24 h. (**c**) ING1b binds to the lincRNA-p21 promoter at ~150–400 bp upstream of the first exon as demonstrated by ING1 chromatin immunoprecipitation (ChIP) of Hs68 cells treated with either GFP or ING1b adenoviruses. Binding of ING1b at the promoters of lincRNA-p21, PCNA (positive control) and Cyclin A (negative control) was detected using regular PCR and qRT-PCR. A parallel IgG ChIP was used as an additional background control. (**d**) ING1b enrichment at the human lincRNA-p21 promoter normalized to IgG in untreated or ADR-treated p53-null and p53-induced H1299 cells, as quantified by regular PCR and qRT-PCR. (**e** and **f**) ING1b-dependent expression of lincRNA-p21 promoter as measured by levels of luciferase activity normalized to *β*-galactosidase activity. Values are relative to untreated or ADR-treated H1299 cells transfected with empty vector controls. All experiments were carried out in triplicates. Asterisks represent significant comparisons (*P*<0.05)

Recent studies indicate that the RNA-binding protein HuR regulates lincRNA-p21 levels by affecting its degradation.^17^ Specifically, HuR decreases lincRNA-p21 by facilitating lincRNA-p21 degradation by the RISC complex. To determine whether ING1b could be acting on lincRNA-p21 levels indirectly by altering HuR expression, we knocked down HuR using siRNAs and looked at lincRNA-p21 levels after ING1b overexpression. We expected that if ING1b could affect HuR then overexpression of ING1b would result in a greater knockdown of HuR *versus* siRNAs alone. However, although knockdown of HuR increased levels of lincRNA-p21 as previously reported, the extent of HuR knockdown was similar irrespective of ING1b expression (Supplementary Figure S2D), and HuR knockdown did not affect levels of p53 transcript in the absence or presence of ING1b (Supplementary Figure S2E). Furthermore, knockdown of HuR decreased levels of lincRNA-p21 in the absence or presence of ING1b (Figures 4a and b), suggesting that effects of ING1b overexpression on lincRNA-p21 occur independently of HuR, consistent with ING1b knockdown increasing lincRNA-p21 half-life (Supplementary Figure S1E).

### ING1b binds in the lincRNA-p21 promoter region

ING proteins act as chromatin regulators that bind the H3K4Me3 histone mark to recruit HAT or HDAC complexes to alter gene expression.^24, 25^ Examining two different cell types by chromatin immunoprecipitation (ChIP) showed that ING1b binds to a promoter region ~150–400 bp upstream of the first lincRNA-p21 exon (Figure 4c). ING1b was also enriched, but to a lesser degree at the PCNA promoter but not at the Cyclin A promoter as reported previously.^10^ Binding efficiency was similar between untreated and ADR-treated p53-null cells but was reduced in p53-induced cells (Figure 4d), suggesting that damage induced levels of p53 may be competing with ING1b for lincRNA-p21 promoter binding. These results indicate that the lincRNA-p21 promoter is bound by, and is, a potential transcriptional target of ING1b. To address this possibility using an independent method, we performed transcription reporter assays where we cloned the predicted lincRNA-p21 promoter (~1400 bp upstream and 200 bp downstream of the first exon^17^) into a luciferase reporter and transfected this plasmid into cells along with *β*-galactosidase to normalize for transfection efficiency. We also included control or ING1b plasmids to determine effects of ING1b on reporter expression. Results showed significant expression of firefly luciferase when ING1b was overexpressed (Figure 4e), and this was further increased in the presence of ADR (Figure 4f). Thus, data from the above assays are consistent with the idea that lincRNA-p21 expression is regulated by both ING1b and p53.

### ING1b requires the NLS, UBD/PBR and LID domains but not the PHD to regulate lincRNA-p21 expression

**Figure 5:**
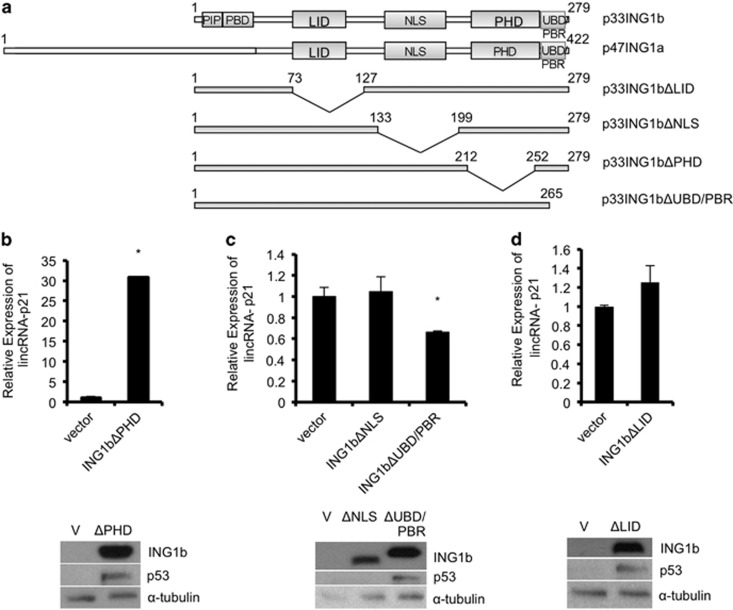
ING1b requires the NLS, UBD/PBR and LID domains to control lincRNA-p21 expression. (**a**) A comparison of ING1 domains and a representation of the ING1b deletion constructs that were used in this study. (**b**, **c** and **d**) Hs68 fibroblasts were treated with either an empty control vector or a deletion mutant of ING1b lacking the plant homeo domain (PHD), nuclear localization signal (NLS), ubiquitin-binding domain/polybasic region (UBD/PBR) or lamin interaction domain (LID) for 48 h before collection of RNA and quantification of lincRNA-p21 expression by qRT-PCR. RNA levels were normalized to GAPDH expression and compared with controls. ING1b, p53 and *α*-tubulin protein expression levels were measured using western blot analysis. All experiments were carried out in triplicate and asterisks indicate *P*<0.05)

The ING1 gene encodes two major variants with ING1b being the dominant isoform in young (low passage) primary cells, whereas ING1a increases significantly at high-passage levels.^10^ To test if ING1a could also affect lincRNA-p21 expression, we overexpressed ING1a and subsequently checked lincRNA-p21 levels. Similar to effects seen with overexpression of ING1b, lincRNA-p21 also increased in response to ING1a overexpression (Supplementary Figure S3A). Interestingly, high-passage (senescent) cells also had high basal levels of lincRNA-p21 (Supplementary Figure S3B). Furthermore, lincRNA-p21 was significantly reduced in HGPS cells, a cell type known to have reduced ING1a and ING1b,^26^ and high levels of p53 expression^27, 28, 29^ (Supplementary Figure S3C). As shown in Figure 5a, the C-terminal region encompassing the lamin interaction domain (LID), nuclear localization sequence (NLS), PHD and the polybasic region/ubiquitin-binding domain (PBR/UBD) of ING1a and ING1b are identical. The PBR, which activates ING1 and ING2 by binding stress-induced phospholipids,^30, 31^ overlaps with the UBD, resulting in competition between ubiquitin and phospholipids for binding of this region.^14^

As the INGs are histone readers that recognize nucleosomes via trimethylated H3K4 through their PHD domains, and the lincRNA-p21 promoter is enriched in with H3K4me3, we asked whether the ING1 PHD domain is required for increasing lincRNA-p21 after ING1b overexpression. To test this, we transfected human fibroblasts with empty control vector or ING1b deletion mutants (ING1bΔPHD, ING1bΔNLS, ING1bΔUBD/PBR or ING1bΔLID as depicted in Figure 5a) and measured the levels of lincRNA-p21. Contrary to our prediction, lincRNA-p21 still increased after expression of the PHD deletion mutant, suggesting that the PHD domain was dispensable (Figure 5b). In contrast, the NLS, UBD/PBR and LID regions were all needed for increasing lincRNA-p21 after ING1b overexpression (Figures 5c and d). Levels of p53 protein were higher after expression of every ING1b mutant with the exception of ING1bΔNLS, consistent with a previous study.^15^ Interestingly, p21, a known target of ING1b and p53 that is found directly downstream of lincRNA-p21 on the DNA, also required the same domains of ING1b to remain intact for proper induction (Supplementary Figures S3D and F), suggesting a common mechanism that does not require recognition of H3K4me3. A similar observation has been made for the ability of ING2 to affect muscle differentiation,^32^ indicating major roles for the ING proteins that are independent of histone mark recognition.

### LincRNA-p21 is an effector of ING1b-mediated apoptosis

**Figure 6:**
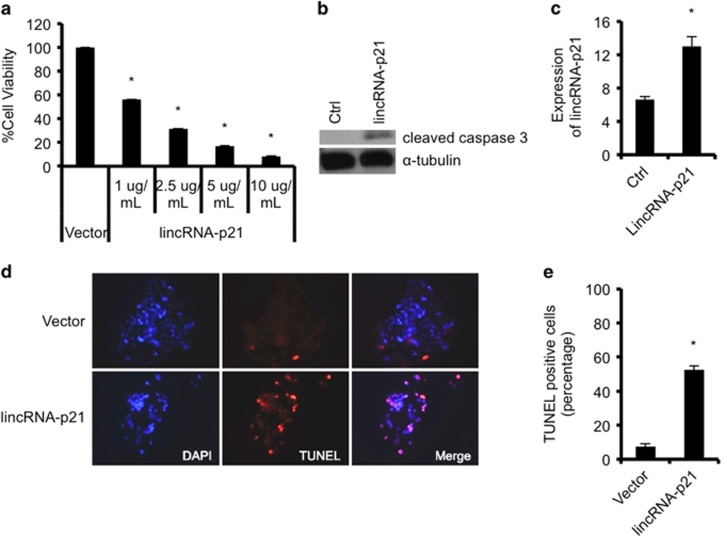
Overexpression of human lincRNA-p21 in cells results in apoptosis. (**a**) Human embryonic kidney cells (HEK293) were transfected with control or lincRNA-p21 plasmids at various concentrations for 24 h, and percentage of cell viability was determined using trypan blue exclusion assays. (**b**) lincRNA-p21 overexpression causes increased caspase 3 cleavage. Cleaved caspase 3 or *α*-tubulin protein expression in HEK293 cells transfected with 1 ug/ml of lincRNA-p21 plasmids for 24 h was assessed using western blot analysis. (**c**) Quantification of lincRNA-p21 expression by qRT-PCR after overexpression of 1 ug/ml of lincRNA-p21 constructs for 24 h in Hep3B cells. (**d** and **e**) Cells in **c** were stained using the TUNEL assay and percentage of TUNEL-positive cells in lincRNA-p21 overexpressed samples were compared with control vector treated cells. RNA levels were normalized to GAPDH expression. Experiments were carried out in triplicates and asterisks indicate *P*<0.05

As ING1b overexpression could induce apoptosis, we next tested if lincRNA-p21, which was also shown to induce apoptosis,^16^ could affect ING1b-mediated cell death. In line with past studies, we found that there was a decrease in cell viability using the trypan blue method, with increasing concentrations of lincRNA-p21 plasmid transfection (Figure 6a). We further measured cleaved caspase 3 proteins as an indicator of apoptosis and found that lincRNA-p21 expression increased caspase 3 cleavage (Figure 6b). Furthermore, transfection of lincRNA-p21 resulted in a greater number of TUNEL-positive cells compared with control (Figures 6c and e), further confirming that lincRNA-p21 can induce apoptosis in human cells.

**Figure 7:**
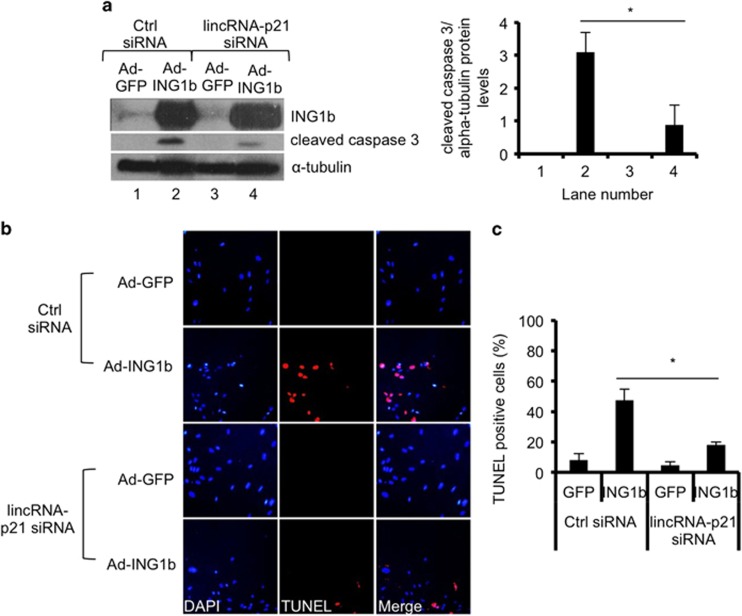
LincRNA-p21 expression affects ING1b-induced apoptosis. Human Hs68 cells were simultaneously transfected with control siRNAs or lincRNA-p21 siRNAs and infected with GFP or GFP-ING1b adenoviruses for 48 h. (**a**) Total proteins were extracted and cleaved caspase 3 levels were measured as an indicator of apoptosis. The plot illustrates quantification of protein blots using ImageJ. Bars represent mean protein expression that was normalized to *α*-tubulin±S.E.M. (asterisks indicate that *P*<0.05, *n*=3). (**b**) Cells were analyzed using the TUNEL assay and immunofluorescence microscopy for apoptosis (red). All fields for each fluor were exposed for the same time. (**c**) Data in graph shows percent of apoptotic cells as shown in panel **b** (values show the mean of three independent replicates±STD, asterisks indicate that *P*<0.05)

If lincRNA-p21 functions downstream of ING1b as suggested, then simultaneous overexpression of ING1b and knockdown of lincRNA-p21 should result in reduced apoptosis compared with ING1b overexpression alone. To test this, we treated normal human cells with control or lincRNA-p21 siRNAs (Figures 7a and c and Supplementary Figure S4A and C) and simultaneously induced ING1b. Subsequently, TUNEL assays were performed and cleaved caspase 3 levels were measured. As observed in Figure 7a and Supplementary Figure S4D, in both human and mouse cells, the increase in cleaved caspase 3 owing to ING1b overexpression in control cells was several-fold higher than in cells with reduced lincRNA-p21. A similar observation was made in cells treated with ADR, albeit the reduction in cleaved caspase 3 caused by lincRNA-p21 knockdown was slightly less than in untreated samples (Supplementary Figure S4D). In addition, as assayed independently using TUNEL assays, ING1b-mediated apoptosis was significantly less efficient when lincRNA-p21 levels were diminished (Figures 7b and c). These results suggest that lincRNA-p21 functions downstream of ING1b and contributes to the induction of apoptosis by ING1b. The correlations between ING1b and lincRNA-p21 noted in this study imply that both components may contribute to anticancer activities of ADR in murine and human cells. This is consistent with both p53 and ING1b individually contributing to ADR-induced apoptosis as noted by TUNEL analysis of Hs68 human diploid fibroblasts (Supplementary Figure S5).

## Discussion

In this study, we show that lincRNA-p21 is induced by ING1. Its effect is additive with p53 and is independent of the PHD domain or the HuR protein that promotes lincRNA-p21 degradation. ING1b also bound to the endogenous lincRNA-p21 promoter region and activated transcription of a lincRNA-p21 promoter-driven reporter construct. Knocking down lincRNA-p21 also reduced the ability of ING1b to induce apoptosis by approximately half, indicating that lincRNA-p21 is a major downstream effector of ING1b.

ING proteins function as readers of the histone code by virtue of their ability to specifically recognize and bind to the H3K4Me3 histone mark via their PHD form of zinc finger. This interaction has been defined both by mutational analyses and at the atomic level by X-ray crystallography.^5, 6, 7, 8^ ING1 and ING2 are stoichometric members of histone deacetylase complexes, specifically the Sin3A complex that contains HDAC1 and HDAC2, and so are thought to target HDAC activity to regions rich in H3K4Me3. This mechanism is thought to be largely responsible for regulating gene expression, as both ING1 and ING2 overexpression results in the repression of more genes than are activated.^33^ LincRNA-p21, which is transcriptionally regulated by p53 and induces apoptosis in response to DNA damage,^16^ is very strongly induced by ING1b in normal diploid cells, and to a lesser extent in established cell lines. This difference may be due to established cell lines having a partially activated but less inducible stress response than normal primary diploid cells.^30^

Our experiments showed that lincRNA-p21 levels positively corresponded with ectopic ING1b expression and ING1b alone was sufficient to increase lincRNA-p21 in p53-null cells. Furthermore, ING1b was necessary for a robust rise in lincRNA-p21 upon exposure of cells to stress using ADR, but not other stresses such as UV and H_2_O_2_. These observations suggest that UV-induced DNA crosslinking and the form of oxidative stress caused by H_2_O_2_ efficiently affect lincRNA-p21 in the absence of ING1, whereas the anthracycline antibiotic ADR (also called doxorubicin) requires ING1 for maximal induction of lincRNA-p21. ADR is a DNA-intercalating agent that poisons toptisomerase II and induces double-stranded breaks. It can also evict histones including the DNA-damage-associated H2AX variant from chromatin, altering the transcriptome and epigenome,^34^ which might be expected to affect epigenetic regulators such as the ING proteins.

**Figure 8:**
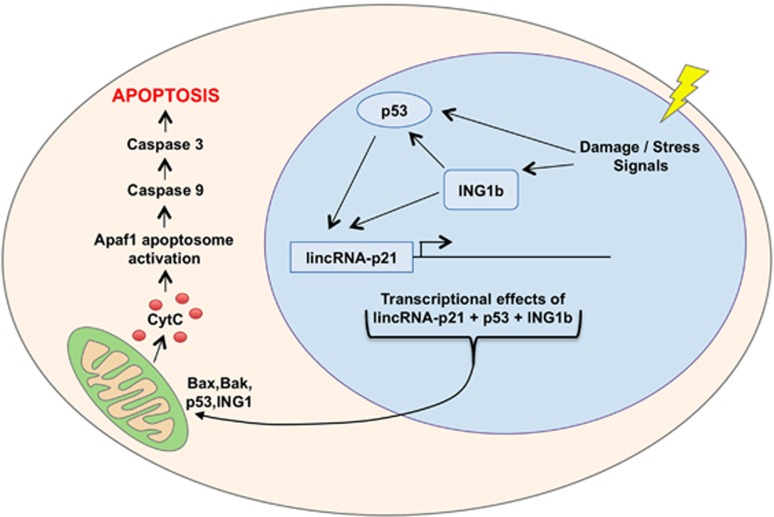
A schematic representation of the functional interdependence between ING1b and p53 in DNA-damage induction of apoptosis. Damage to DNA or to other macromolecules initiates a stress response that activates ING1b by phosphoinositide binding and stabilizes p53 by ING1-dependent and -independent pathways that inhibit p53 ubiquitination. ING1 and p53 then converge at the level of lincRNA-p21, inducing an increase in its expression. Subsequently, lincRNA-p21 reinforces transcriptional changes by p53 and ING1b that regulate Bax and Bak expression, which then converge with both p53 and ING1b at the mitochondrial membrane to promote release of cytochrome C. Variable amounts of ING1b and p53 localize to the mitochondrial membrane, depending upon cell type. Cyt C then induces assembly of Apaf 1 into active apoptosomes, initiating the dimerization and processing of procaspase 9 on the apoptosome. Active caspase 9 then cleaves procaspase 3 to produce active caspase 3 and the initiation of proteolysis inducing apoptosis

The observations noted above indicate that both ING1 and p53 contribute approximately equally to the induction of lincRNA-p21. We found that ING1b bound the lincRNA-p21 promoter and drove the expression of reporter constructs, whereas a previous study noted the presence of p53-binding sites in the same region, consistent with ING1 and p53 jointly inducing lincRNA-p21 transcription. A model highlighting this relationship is shown in Figure 8. Knockdown of lincRNA-p21 also showed that it contributed significantly to ING1b-induced apoptosis. However, the following two observations do not fit simply into this model: ING1b binding to the lincRNA-p21 promoter occurs most efficiently when p53 is lacking in the cell suggesting that ING1b and/or p53 also affect lincRNA-p21 expression by mechanisms other than those requiring promoter binding. In addition, the PHD region of ING1, which binds chromatin at HeK4Me3 marks, is not required for induction, further suggesting that ING1 contributes to induction of lincRNA-p21 by means other than affecting nucleosome structure through binding H3K4Me3. ING1b is not a transcriptional target of p53^32^ but rather appears to protect p53 from degradation^15, 35^ to enhance its activity. This represents another mechanism by which ING1b and p53 may interact to increase levels of lincRNA-p21, which might also explain their convergence of function in inducing apoptosis, an observation made independently in previous studies.^1, 10, 11, 12^

## Materials and methods

### Overexpression of ING1 variants, lincRNA-p21, p53 induction and DNA damage

To overexpress ING1 wild-type proteins and deleted mutants, cultured cells were transfected with pCI-ING1bΔPHD/ pcDNA-ING1bΔLID/ pEGFP.C2-ING1bΔNLS/ pEGFP.C2-ING1bΔUBD/ PBR plasmids or treated with adenoviruses containing GFP-ING1 for 24–48 h at a multiplicity that yielded 95% infection efficiency. Overexpression of human lincRNA-p21 was accomplished using pMS2-lincRNA-p21 plasmids (kind gifts from Dr Myriam Gorospe at the National Institutes of Health, Baltimore, MD, USA).^17^ All plasmid transfections were carried out using Lipofectamine LTX (Invitrogen, Burlington, ON, Canada). Cells infected with Ad-GFP or transfected with appropriate empty vectors were used as controls. For induction of p53, H1299 cells harboring an inducible p53 expression construct (a kind gift of Dr Patrick Lee at Dalhousie University, Halifax, Nova Scotia, Canada) were treated with 100 ng/ml of doxycycline for 24 h. For DNA damage, cells were treated with 400–500 nM ADR (Sigma D1515, Oakville, ON, Canada) for 16 h, 100 *μ*M H_2_O_2_ for 24 h (Sigma), and exposed to 85 J/m^2^ of UV (CL-1000 UV Crosslinker) and harvested at 24 h.

### RNA interference

Knockdown experiments were performed using siRNA oligos that target the genes of interest. The custom siRNAs used were as follows: human p53 (5′-GACUCCAGUGGUAAUCUACtt-3′), human lincRNA-p21 (5′-UCAUCAUGCGGCCUUGCAGtt-3′)^17^ human HuR (5′-CGUAAGUUAUUUCCUUUAAtt-3′),^17^ and mouse lincRNA-p21 (no.1 5′-UGAAAAGAGCCGUGAGCUA-3′, no. 2 5′-AAAUAAAGAUGGUGGAAUG-3′ and no. 3 5′-AGUCAAAGGCAAUGAGCAU-3′).^16^ ING1 and non-targeting siRNAs were purchased from Dharmacon (Ottawa, ON, Canada) (L-006533 and D-001206-14, respectively). All transfections were carried out with 100 nM of siRNAs using Lipofectamine 2000 (Invitrogen) according to the manufacturer's instructions.

### RNA extraction and preparation

RNA was isolated from cells using Trizol (Invitrogen) and purified using the RNeasy Mini Kit (Qiagen). The purity and concentration of the RNA were assessed using a Nanodrop ND-1000 (Thermo Scientific, Waltham, MA, USA) as per the manufacturer's instructions.

### Quantitative real-time polymerase chain reaction

Reverse transcription was conducted to create templates for qRT-PCR using the High-Capacity cDNA synthesis kit (Applied Biosystems, Foster City, CA, USA) or the iScript cDNA synthesis kit (Bio-Rad, Mississauga, ON, Canada). Quantitative real-time PCR was performed using a SYBR green master mix containing 10 × PCR buffer, 50 mM MgCl_2_, 10 mM dNTPs, 10 × SYBR Green (diluted 1:1000), ROX passive reference dye, nuclease-free water and platinum Taq DNA Polymerase (Invitrogen). For RNA expression measurements, both the standard curve and the ΔΔCt methods were performed using the 7900HT thermal cycler system from Applied Biosystems. The human primers used in this study are listed in Supplementary Table S1. GAPDH, beta-actin, 18 S rRNA and histone 3 (h3) RNA levels were used for normalization.

### Chromatin immunoprecipitation

Hs68 cells were treated with either GFP or ING1b adenoviruse for 24 h. H1299 cells were transfected with pCI-ING1b. Eight hours after transfection, 100 ng/ml doxycycline was added for 16 h. Subsequently, cells were treated with 500 nM ADR for an additional 16 h. All cells were harvested using trypsinization, and ChIP was performed as previously described.^36^ In brief, cells were crosslinked with 1% formaldehyde, harvested, resuspended in lysis buffer that was supplemented with 1 mM PMSF, and sonicated. The chromatin was sheered to an average size of 500–1000 bp and immunoprecipitated using either ING1^37^ or IgG (Chemicon, Etobicoke, ON, Canada) background control antibodies. After extensive washing steps, the formaldehyde crosslinks were reversed by treatment at 65 ^°^C. DNA was purified by phenol/chloroform extraction followed by ethanol precipitation and samples were used in semi-quantitative PCR (primers are shown in Supplementary Table S1).

### Cloning and promoter reporter assays

The human lincRNA-p21 promoter region was cloned into the pGL3-basic vector (Promega, Madison, WI, USA). H1299 cells containing a doxycycline-inducible p53 promoter were transfected with either pCI or pCI-ING1b in conjunction with 100 ng of pGL3-lincRNA-p21 and 25 ng of pCMV-*β*-galactosidase in a 24-well plate. Eight hours post transfection, the cells were treated with 100 ng/ml doxycycline. At 24 h, cells were treated with 500 nM of ADR for an additional 8 h. Cells were then washed with 1 × PBS, lysed with reporter lysis buffer (Promega E397a) and left at −20 ^o^C overnight. Cell extracts were assayed for luciferase and *β*-galactosidase activity the following day.

### Viability assays

Viability of Hep3B cells was assessed using Trypan Blue exclusion assays. In brief, cells were collected by trypsinization after transfection with either control vector or pMS2-lincRNA-p21 plasmids and stained with 0.4% trypan blue reagent (Sigma). The number of live cells was determined using a hemocytometer. All experiments were performed in triplicate.

### Immunofluorescence microscopy (TUNEL assay)

Cells were seeded into eight-chamber culture slides (BD Biosciences, Mississauga, ON, Canada) where they were transfected with either control or lincRNA-p21 siRNAs, and infected with adeno-GFP or adeno-ING1b viruses for a total of 48 h. Subsequently, TUNEL assays were performed using the ApopTag Red *In Situ* Apoptosis Detection Kit (Millipore, Etobicoke, ON, Canada) according to the manufacturer's recommendations. Post processing, the cells were visualized using a Zeiss Axiovert 200 microscope and images were captured using AxioVision software.

## Abbreviations

ActD: actinomycin D
ADR: adriamycin/doxorubicin
bp: base pairs
ChIP: chromatin immunoprecipitation
CMV: cytomegalovirus
GAPDH: glyceraldehyde-phosphate dehydrogenase
GFP: green fluorescent protein
H2AX: histone H2A variant X
H3K4Me3: trimethylated lysine 4 of histone H3
HAT: histone acetyltransferase
HDAC: histone deacetylase
HuR: RNA-binding protein human antigen R
ING1b: inhibitor of growth 1 isoform b
ING2: inhibitor of growth 2
KO: knockout
LID: lamin interaction domain
lincRNA-p21: large intergenic non-coding ribonucleic acid-p21
lncRNA: long non-coding RNA
MEF: mouse embryo fibroblast
NLS: nuclear localization sequence
p21: 21 kilodalton cyclin-dependent kinase inhibitor
p53: 53 kilodalton tumor suppressor protein
PBR: polybasic region
PBS: phosphate-buffered saline
PHD: plant homeodomain form of zinc finger
PCNA: proliferating cell nuclear antigen
qRT-PCR: quantitative real time-polymerase chain reaction
RISC: RNA-induced silencing complex
siRNA: silencing RNA
TUNEL: terminal deoxynucleotidyl transferase nick end labelling
UBD: ubiquitin binding domain that overlaps the PBR
UV: ultra violet light
WT: wild-type
