# Supplementary material for: LincRNA-p21 acts as a mediator of ING1b-induced apoptosis
Source: Cell Death Dis. 2015 Mar 5;6(3):e1668–. doi: 10.1038/cddis.2015.15 (PMC4385912; doi:10.1038/cddis.2015.15)
Supplement: Supplementary Information [file cddis201515x1.doc]

**LincRNA-p21 acts as a mediator of ING1b-induced apoptosis**

Uyen M. Tran^1^, Uma Rajarajacholan^1^, Jung Soh^2^, Tae-sun Kim^1^, Subhash Thalapilly^1^, Christoph W. Sensen^2,3^ and Karl Riabowol^1^,*

^1^ Department of Biochemistry and Molecular Biology, and Oncology, University of Calgary, 3330 Hospital Drive NW, Calgary, Alberta, T2N 4N1, Canada

^2^ Visual Genomics Centre, Department of Biochemistry and Molecular Biology, University of Calgary, 3330 Hospital Drive NW, Calgary, Alberta, T2N 4N1, Canada

^3^ current address: Graz University of Technology, Institute of Molecular Biotechnology, Petersgasse 14, A-8010 Graz, Austria

**SUPPLEMENTARY MATERIALS AND METHODS**

**Cell culture**

Human foreskin diploid fibroblasts (Hs68, MPD 30-50; ATCC) and A431 cells used in this study were grown in low glucose Dulbecco’s Modified Eagle Medium (DMEM). Human primary HGPS fibroblasts (AG11513 and AG00989; Coriell Cell Repositories) and human hepatocellular carcinoma cells (HEP3B) were maintained in Eagle’s Minimum Essential Medium (MEM) with the addition of 2 mmol/L of L-glutamine. Human non-small cell lung cancer cells (H1299; kind gifts from Dr. Patrick Lee at Dalhousie University, Halifax, Nova Scotia, Canada) were cultured with RPMI-1640 media. Mouse embryonic fibroblasts (MEF) were grown in high glucose DMEM with the addition of sodium pyruvate. All media were supplemented with 10% fetal bovine serum (FBS) with the exception of HGPS cells, which were grown with 15% FBS. All cells were maintained at 37^o^C with 21% oxygen and 5% carbon dioxide in a humidified incubator.

**Protein Extraction**

Total proteins were extracted using a non-denaturing cell lysis buffer containing 20 mM Tris-HCl (pH 7.5), 150 mM NaCl, 1 mM Na_2_EDTA, 1 mM EGTA, 1% NP-40, 1% sodium deoxycholate, 2.5 mM sodium pyrophosphate, 1 mM beta-glycerophosphate, 1 mM Na_3_VO_4_ and 1 µg/ml leupeptin (Cell Signaling Technology #9803). In order to inhibit proteases, an addition of 1 mM phenylmethylsulfonyl fluoride (PMSF) was also added to the solution prior to the extraction. Then, the proteins were dyed using the Bradford protein assay (Bio-Rad) and their concentrations were measured using a spectrophotometer. Total proteins were diluted in 1X Laemmli sample buffer and boiled for 5 minutes at 95 ^o^C before electrophoresis.

**Western Blotting**

SDS-PAGE was conducted using the Mini Trans-Blot® Electrophoretic Transfer Cell system from Bio-Rad (#170-3930). Thirty micrograms of total proteins from each sample were resolved into different molecular weight bands on 12% poly-acrylamide gels in running buffer at 100-120 V for 1-2 hours. Proteins were then transferred onto nitrocellulose membranes and blocked for 1 hour at room temperature using 5% non-fat skim milk in PBS-T (0.1% Tween-20). The blots were then incubated with various primary antibodies overnight at 4 ^o^C: anti-ING1 antibody (SACRI Antibody Services), anti-p53 anbibody (SACRI Antibody Services), anti-α-tubulin at 1:200 dilution (Santa Cruz Biotechnology #sc-8035), and anti-cleaved caspase 3 antibody at 1:1000 dilution (Cell Signaling Technology #9661). Subsequent to washing steps using PBS-T, membranes were incubated in goat anti-mouse IgG or goat anti-rabbit horseradish peroxidase-linked secondary antibodies at 1:10,000 dilution (Chemicon) for 30-45 minutes at room temperature. With the exception of anti-ING1 and anti-p53 antibodies, which were acquired in complete serum and added directly to the membranes, all other antibodies were diluted using 5% non-fat skim milk in PBS-T (0.1% Tween-20). Following additional washing steps, antibody binding was detected by adding Immobilon western chemiluminescent HRP substrate (Millipore) onto the membranes for 1 minute, exposing them to X-ray films and then developing the films using a Kodak X-OMAT 2000 processor. For re-probing, membranes were stripped in 0.5 M NaOH for 3 minutes, washed and regular western blotting methods were performed. For statistical analysis, protein bands were quantified using Image J and normalized to loading controls (α-tubulin).

**Overall statistical analysis**

Unless indicated otherwise, all results are the mean ± S.E.M of at least three independent experiments. Data analyses were done using an unpaired student’s T-test. Asterisks indicate a p-value of <0.05 and was considered as statistically significant.

**SUPPLEMENTARY REFERENCES**

1. Huarte, M., Guttman, M., Feldser, D., Garber, M., Koziol, M.J., Kenzelmann-Broz, D., Khalil, A.M., Zuk, O., Amit, I., Rabani, M. *et al.* (2010) A large intergenic noncoding RNA induced by p53 mediates global gene repression in the p53 response. *Cell*, **142**, 409-419.

2. Soliman, M.A., Berardi, P., Pastyryeva, S., Bonnefin, P., Feng, X., Colina, A., Young, D. and Riabowol, K. (2008) ING1a expression increases during replicative senescence and induces a senescent phenotype. *Aging Cell*, **7**, 783-794.
