# Supplementary figures and images for: LincRNA-p21 acts as a mediator of ING1b-induced apoptosis
Source: Cell Death Dis. 2015 Mar 5;6(3):e1668–. doi: 10.1038/cddis.2015.15 (PMC4385912; doi:10.1038/cddis.2015.15)

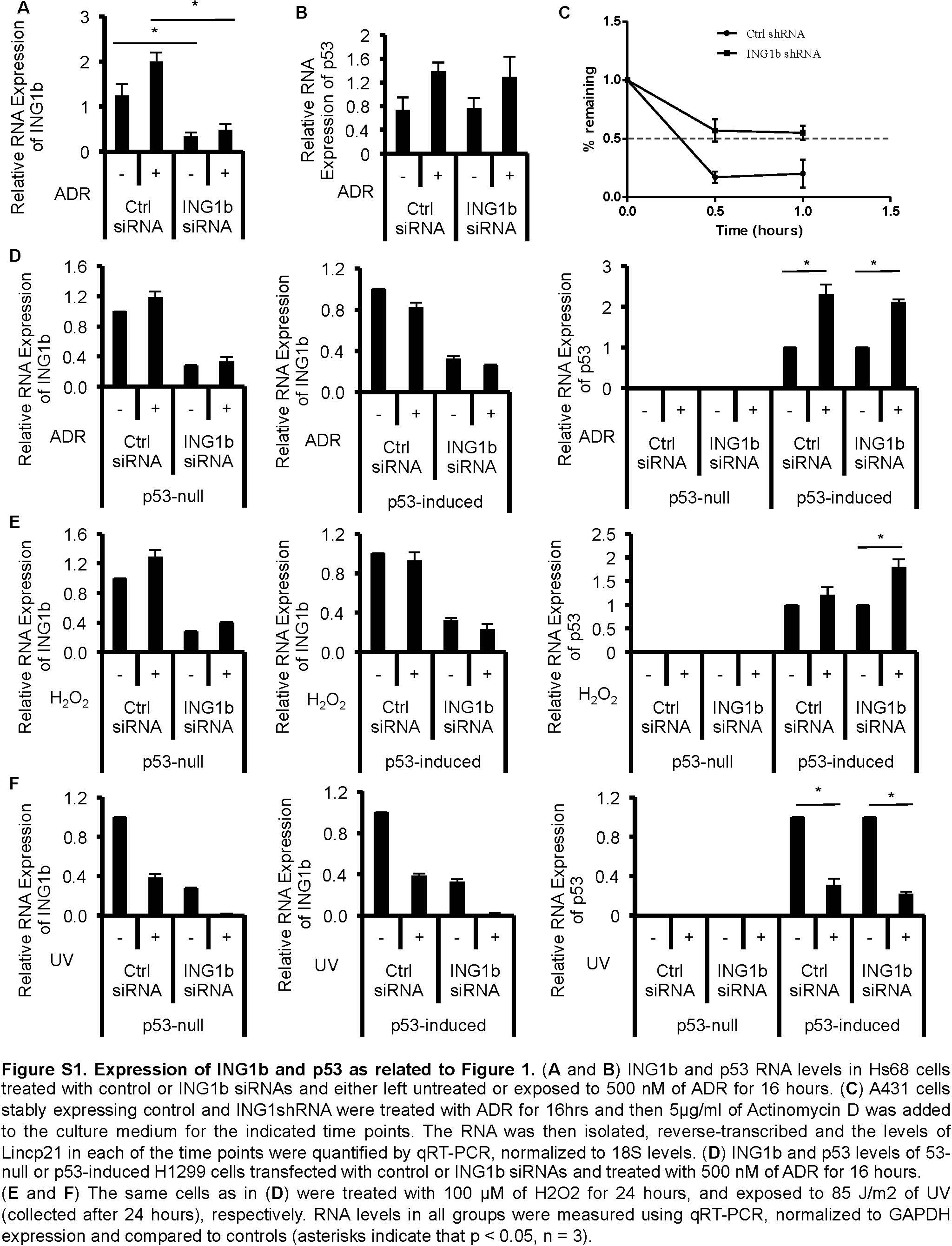

Supplement: Supplementary Figure 1 [file cddis201515x2.tif]

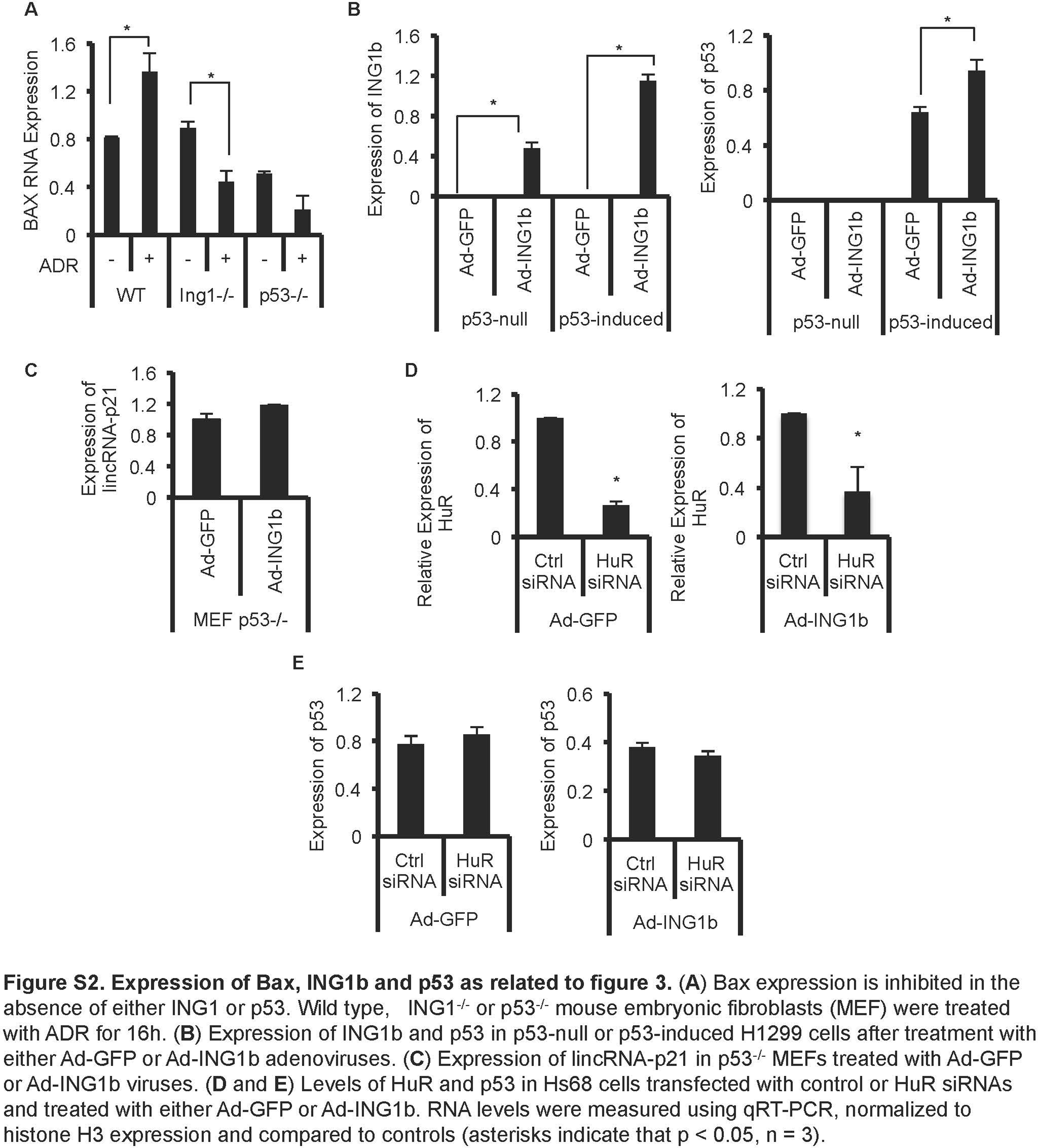

Supplement: Supplementary Figure 2 [file cddis201515x3.tif]

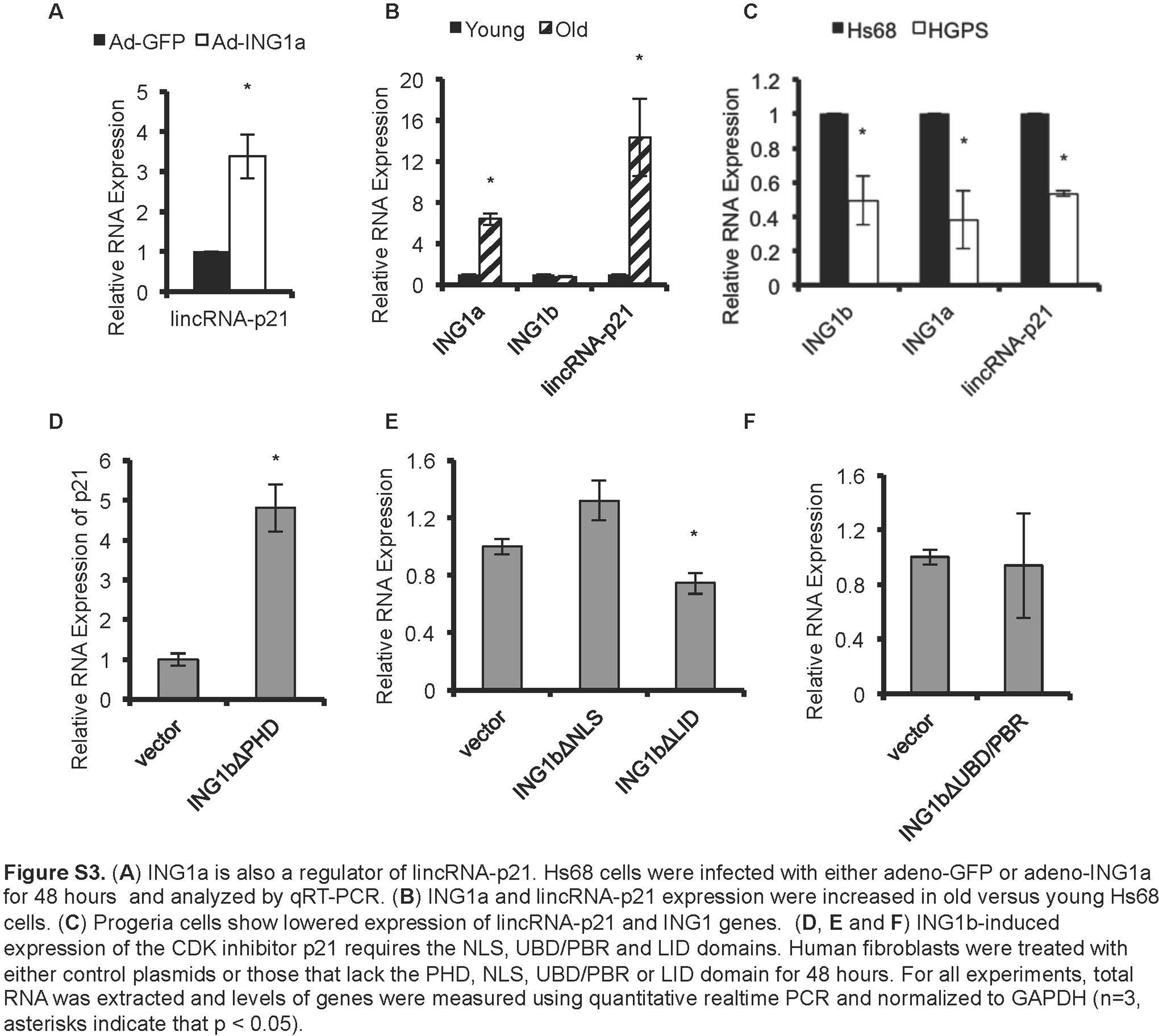

Supplement: Supplementary Figure 3 [file cddis201515x4.tif]

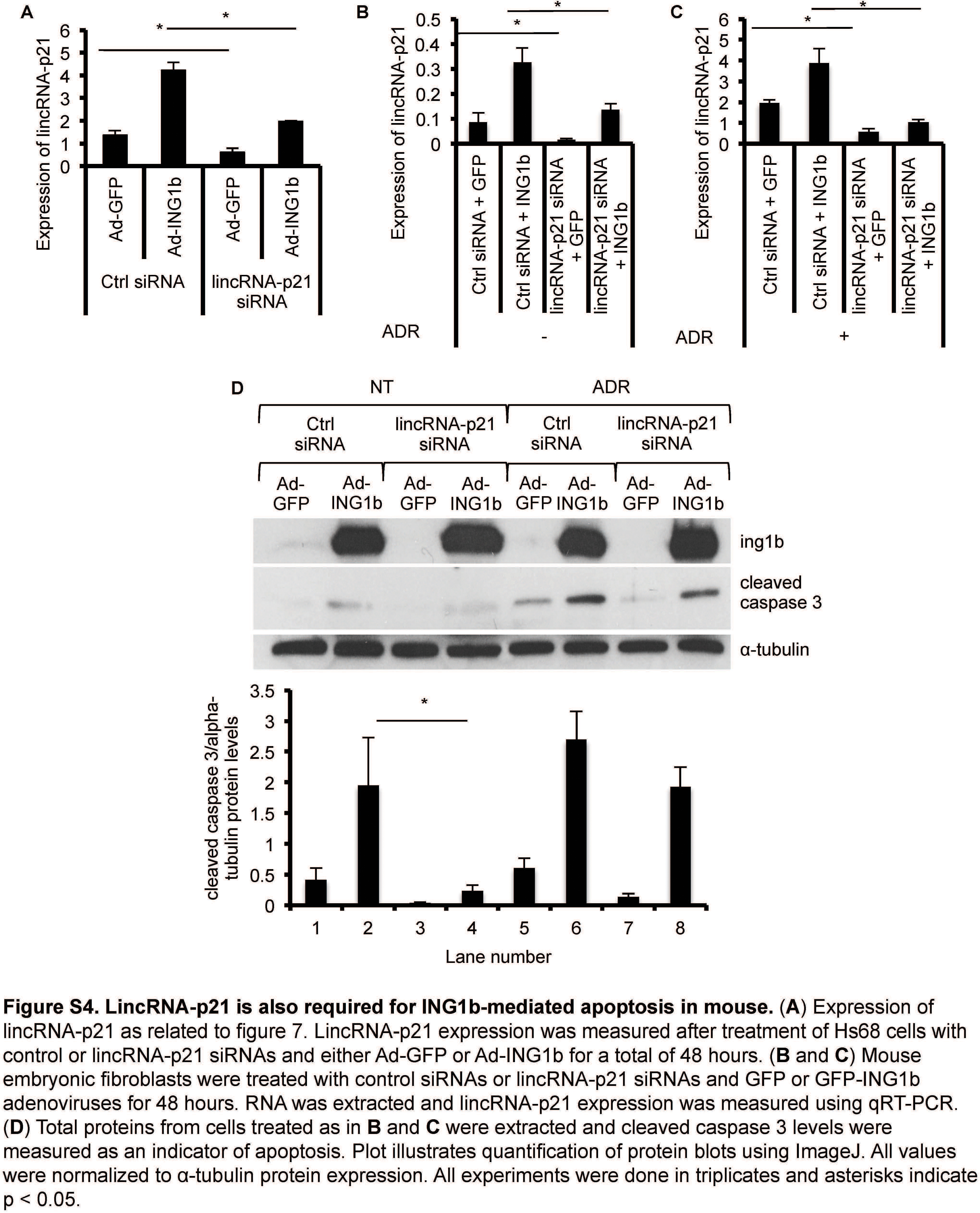

Supplement: Supplementary Figure 4 [file cddis201515x5.tif]

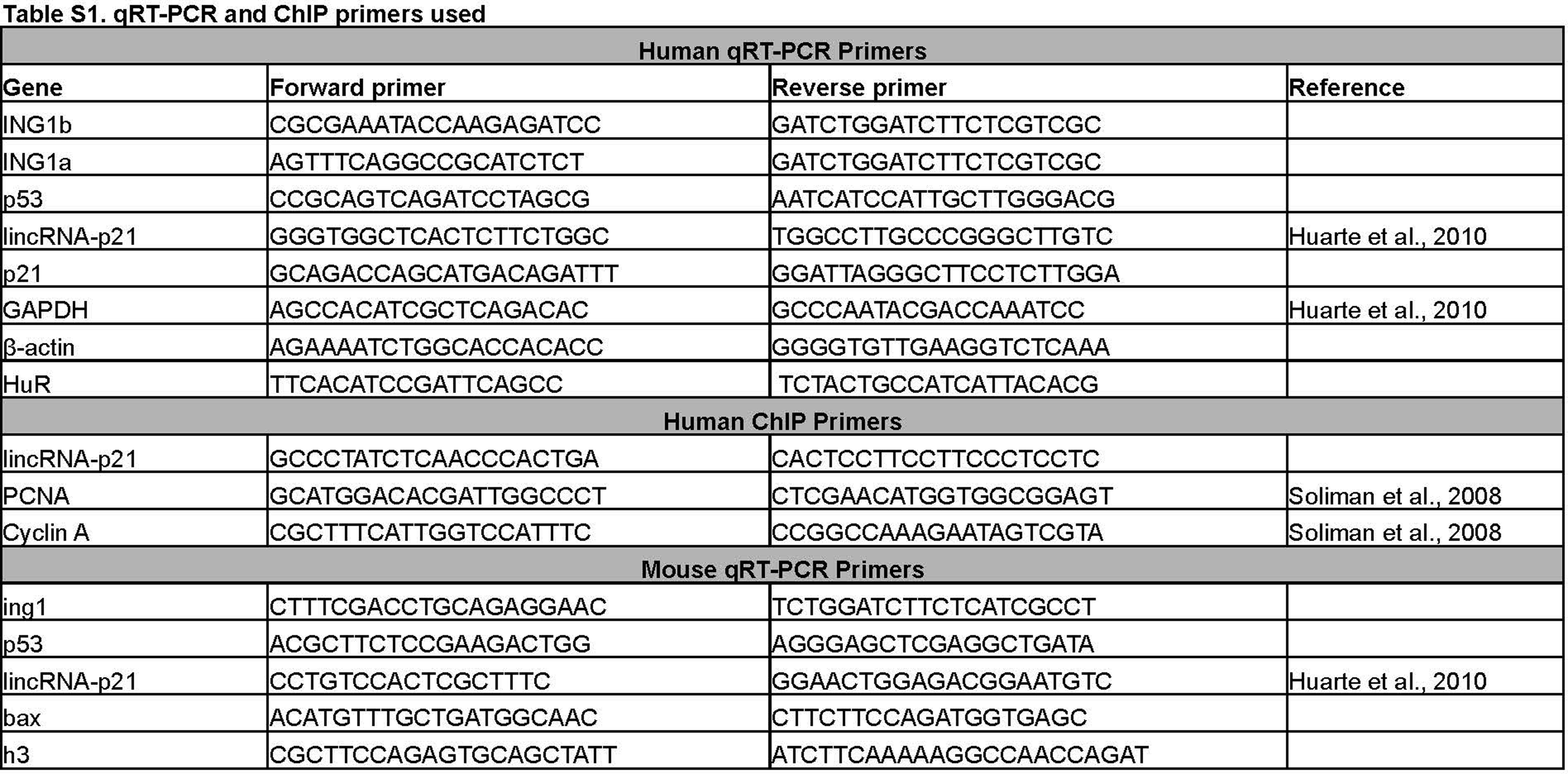

Supplement: Supplementary Table 1 [file cddis201515x7.tif]
